# Primary Cilia Formation Does Not Rely on WNT/β-Catenin Signaling

**DOI:** 10.3389/fcell.2021.623753

**Published:** 2021-02-26

**Authors:** Ondrej Bernatik, Petra Paclikova, Anna Kotrbova, Vitezslav Bryja, Lukas Cajanek

**Affiliations:** ^1^Department of Histology and Embryology, Faculty of Medicine, Masaryk University, Brno, Czechia; ^2^Section of Animal Physiology and Immunology, Department of Experimental Biology, Faculty of Science, Masaryk University, Brno, Czechia

**Keywords:** primary cilia, Wnt/β-catenin, ciliogenesis, cell signaling, Wnt3a, RPE-1, HEK293, NIH3T3

## Abstract

Primary cilia act as crucial regulators of embryo development and tissue homeostasis. They are instrumental for modulation of several signaling pathways, including Hedgehog, WNT, and TGF-β. However, gaps exist in our understanding of how cilia formation and function is regulated. Recent work has implicated WNT/β-catenin signaling pathway in the regulation of ciliogenesis, yet the results are conflicting. One model suggests that WNT/β-catenin signaling negatively regulates cilia formation, possibly via effects on cell cycle. In contrast, second model proposes a positive role of WNT/β-catenin signaling on cilia formation, mediated by the re-arrangement of centriolar satellites in response to phosphorylation of the key component of WNT/β-catenin pathway, β-catenin. To clarify these discrepancies, we investigated possible regulation of primary cilia by the WNT/β-catenin pathway in cell lines (RPE-1, NIH3T3, and HEK293) commonly used to study ciliogenesis. We used WNT3a to activate or LGK974 to block the pathway, and examined initiation of ciliogenesis, cilium length, and percentage of ciliated cells. We show that the treatment by WNT3a has no- or lesser inhibitory effect on cilia formation. Importantly, the inhibition of secretion of endogenous WNT ligands using LGK974 blocks WNT signaling but does not affect ciliogenesis. Finally, using knock-out cells for key WNT pathway components, namely DVL1/2/3, LRP5/6, or AXIN1/2 we show that neither activation nor deactivation of the WNT/β-catenin pathway affects the process of ciliogenesis. These results suggest that WNT/β-catenin-mediated signaling is not generally required for efficient cilia formation. In fact, activation of the WNT/β-catenin pathway in some systems seems to moderately suppress ciliogenesis.

## Introduction

Primary cilia are tubulin-based rod-shaped organelles on the surface of most mammalian cells. They play a fundamental role in embryo development and tissue homeostasis. Importantly, defects in primary cilia structure and function lead to variety of developmental disorders collectively called ciliopathies (Hildebrandt et al., 2011; Mitchison and Valente, 2017; Reiter and Leroux, 2017). Moreover, primary cilia defects have been related to cancer (Han et al., 2009; Wong et al., 2009; Jenks et al., 2018).

Cilium formation is organized by the mother centriole (MC)-derived basal body, the older centriole of the pair that makes up the centrosome. While centrosome is best known as microtubule organizing center coordinating mitosis, primary cilium formation is tightly connected with G1/G0 phase (Ford et al., 2018; Mirvis et al., 2018). The growth of primary cilium itself is preceded by the accumulation of vesicles at MC distal appendages (Sorokin, 1962; Westlake et al., 2011; Schmidt et al., 2012; Lu et al., 2015; Wu et al., 2018) and by the removal of CEP97/CP110 capping complex specifically from MC distal end (Spektor et al., 2007). Major role in the cilia initiation is linked to the Tau tubulin kinase 2 (TTBK2) activity (Goetz et al., 2012). Once recruited to MC by distal appendage protein CEP164 (Čajánek and Nigg, 2014; Oda et al., 2014), TTBK2 seems to control both the process of vesicle docking and the CP110/CEP97 removal (Goetz et al., 2012; Lo et al., 2019). In turn, this allows the extension of tubulin-based axoneme sheathed by ciliary membrane from MC-derived basal body. The formed cilium is physically separated from the rest of a cell by ciliary transition zone, a selective barrier ensuring only specific proteins to enter the cilium (Garcia-Gonzalo and Reiter, 2017; Gonçalves and Pelletier, 2017; Nachury, 2018). Such compartmentation and hence specific protein composition of primary cilium is the basis for its instrumental role in the Hedgehog signaling pathway in vertebrates (Bangs and Anderson, 2017; Nachury and Mick, 2019). In addition, several links between primary cilia and other signaling pathways such as WNT or TGF-β have recently emerged (Anvarian et al., 2019).

WNT signaling pathways are developmentally important signaling routes regulating cell differentiation, migration, and proliferation and their activity controls shaping of the embryo (Nusse and Clevers, 2017). WNT signaling pathways can be distinguished based on whether they use β-catenin as an effector protein. The pathway relying on stabilization of β-catenin is termed the WNT/β-catenin pathway and regulates stemness, cell differentiation and proliferation, while the β-catenin-independent or non-canonical WNT pathways regulate cytoskeleton, cell polarity, and cell movements (Humphries and Mlodzik, 2018; Steinhart and Angers, 2018). These two branches of WNT pathways are activated by a distinct set of extracellularly secreted WNT ligand proteins (Angers and Moon, 2009). WNTs are posttranslationally palmitoylated by O-Acyl-transferase Porcupine, and only after the lipid modification are the WNT proteins fully active (Willert et al., 2003; Zhai et al., 2004). Following their secretion, WNTs bind to seven-pass transmembrane receptors from Frizzled family that form heterodimeric complexes with various coreceptors. WNT/β-catenin pathway uses LRP5/6 coreceptors (Pinson et al., 2000; Tamai et al., 2000; Wehrli et al., 2000). Signal received by the receptor-coreceptor pair on the cell membrane is then relayed to Dishevelled (DVL) proteins that, following phosphorylation by CK1-δ/ε and other kinases (Bernatik et al., 2011; González-Sancho et al., 2013; Hanáková et al., 2019), are used both by the non-canonical and the WNT/β-catenin pathways (Sokol, 1996; Wallingford et al., 2000). β-catenin destruction complex, composed of proteins Adenomatous polyposis coli (APC), AXIN and two kinases; GSK3-β and CK1-α, is then inactivated by DVL sequestration of AXIN proteins (Tamai et al., 2004). Then β-catenin phosphorylation by GSK3-β and CK1-α on its N-terminal degron is terminated and the non-phosphorylated Active β-catenin (ABC) accumulates, translocates to the nucleus where it binds transcription factors of TCF-LEF family to trigger transcription of target genes (Behrens et al., 1996; Molenaar et al., 1996). Not surprisingly, many developmental disorders and cancers are directly caused by WNT pathways deregulation (Zhan et al., 2017; Humphries and Mlodzik, 2018).

Whilst the connections between primary cilia and hedgehog signaling are well documented (Huangfu et al., 2003; Corbit et al., 2005; Rohatgi et al., 2007), the relationship between cilia and WNT signaling is still rather controversial. The exception here seems to be the WNT/PCP pathway [one of the non-canonical WNT pathways (Butler and Wallingford, 2017)], which was described to affect cilia formation and functions via effects on cytoskeleton and basal body positioning (Wallingford and Mitchell, 2011; May-Simera and Kelley, 2012; Carvajal-Gonzalez et al., 2016; Bryja et al., 2017). As for the WNT/β-catenin pathway, there are reports showing that primary cilia loss or disruption leads to upregulation of the pathway activity (Corbit et al., 2008; McDermott et al., 2010; Wiens et al., 2010; Lancaster et al., 2011; Liu et al., 2014; Zingg et al., 2018; Patnaik et al., 2019), but also studies that deny any involvement of primary cilia in WNT/β-catenin signaling (Huang and Schier, 2009; Ocbina et al., 2009). Some of these discrepancies can perhaps be explained by context-specific activity of involved ciliary components (Lancaster et al., 2011; Patnaik et al., 2019) or effects directly on WNT/β-catenin pathway independently of the role in cilia formation (Balmer et al., 2015; Kim et al., 2016), or the requirement for intact basal bodies rather than cilia (Vertii et al., 2015; Vora et al., 2020).

To make the matters even more puzzling, two opposing models have recently emerged regarding possible function of WNT/β-catenin pathway in cilia formation. Activation of the WNT/β-catenin pathway in neural progenitors of the developing cerebral cortex was reported to hamper cilia formation in mice (Nakagawa et al., 2017), arguing for a negative role of the excesive WNT/β-catenin signaling in ciliogenesis. In contrast, a recent report described a direct involvement of WNT/β-catenin signaling pathway in promotion of primary cilia formation through β-catenin driven stabilization of centriolar satellites in RPE-1 cell line (Kyun et al., 2020). We approached this conundrum using cell lines that commonly serve as ciliogenesis model systems (RPE-1, NIH3T3, and HEK293). Using either pharmacological or genetic means to manipulate the WNT/β-catenin pathway, we found no evidence of facilitated ciliogenesis in response to the activation of WNT/β-catenin signaling.

## Materials and Methods

### Cell Culture

RPE-1 cells were grown in DMEM/F12 (Thermo Fisher Scientific, 11320033) supplemented by 10% FBS (Biosera, cat. No. FB-1101/500), 1% Penicillin/Streptomycin (Biosera, cat. No. XC-A4122/100) and 1% L-glutamine (Biosera, cat. No. XC-T1715/100), HEK293 T-Rex (referred to as HEK293, cat.no. R71007, Invitrogen) and NIH3T3 cells were grown in DMEM Glutamax^®^ (Thermo Fisher Scientific, 10569069) supplemented by 10% FBS and 1% Penicillin/Streptomycin. Where indicated, RPE-1 cells were starved by serum free medium, NIH3T3 cells were starved by 0.1% FBS containing medium, and HEK293 cells were starved by serum free medium for 24 h. Cells were seeded at 50,000/well (RPE-1 and NIH3T3) or 120000/well (HEK293) of 24 well plate. Treatments by small molecules were done for indicated times: LGK974 (0.4 μM) (Sellcheck, cat. No. S7143) for 72 h (LGK974 was re-added to the starvation medium as indicated in Figure 2A), Cytochalasin D (500nM) (Merck Cat. No. C8273) for 16 h, PF670462 (1 μM) (Merck, SML0795) for 24 h. WNT3a (90 ng/ml) (R&D systems, Cat.no. 5036-WN) for 2 h or 24 h.

### Western Blot and Quantification

Western blot was performed as previously described (Bernatik et al., 2020). Antibodies used: LRP6 (Cell signaling, Cat.no. #2560), Phospho-LRP5/6 (Ser1493/Ser1490; Cell signaling, Cat.no. #2568), AXIN1 (Cell signaling, Cat.no. #3323) DVL2 (Cell signaling, Cat.no. #3216), Active-β-catenin (Merck, Cat. no. 05-665-25UG), and α-tubulin (Proteintech, Cat.no. 66031-1-Ig). Quantifications were performed using Fiji distribution of ImageJ. Intensity of pLRP5/6 and ABC band was measured and normalized to mean value from all conditions of given experiment. Intensity of LRP6 and DVL2 was calculated as the ratio of the upper to lower band intensity (the bands are indicated by arrows in the corresponding Figures) and normalized to mean value from all conditions of given experiment. Quantification was performed on *n* = 3. Statistical analyses by students *t*-test or one-way ANOVA were performed using Graphpad Prism, *P* < 0.05 (^∗^), *P* < 0.01 (^∗∗^), *P* < 0.001 (^∗∗∗^), and *P* < 0.0001 (^****^).

### Immunocytochemistry

RPE-1, NIH3T3 and HEK293 cells were seeded on glass coverslips, treated as indicated, washed by PBS and fixed for 10 min in −20^*o*^C methanol, washed 3× by PBS, blocked (2% BSA in PBS with 0.01% NaN_3_), 3× washed by PBS, incubated with primary antibodies for 1 h, 3× washed by PBS, incubated with secondary antibodies (Goat anti-Rabbit IgG Alexa Fluor 488 Secondary Antibody, Cat.no. A11008; Goat anti-Mouse IgG Alexa Fluor 568 Secondary Antibody, Cat.no. A11031, all from Thermo Fisher Scientific) for 2 h in dark, washed 3× by PBS, incubated 5 min with DAPI, 2× washed by PBS and mounted to glycergel (DAKO #C0563). Microscopy analysis was done using Zeiss AxioImager.Z2 with Hamamatsu ORCA Flash 4.0 camera, 63× Apo oil immersion objective, and ZEN Blue 2.6 acquisition SW (Zeiss). Image stacks acquired using Zeiss AxioImager.Z2 were projected as maximal intensity images by using ImageJ distribution FIJI (Schindelin et al., 2012). Where appropriate, contrast and/or brightness of images were adjusted by using Photoshop CS5 (Adobe) or FIJI. To assess effects on ciliogenesis or cilia length, at least 4–5 fields of vision (approximately 200–400 cells per experiment) were analyzed per experimental condition, on at least *n* = 3. Cilia present on HEK293 cells were counted manually. Cilia present on RPE-1 or NIH3T3 were counted in ACDC software semiautomatic mode, all cilia present were verified and adjusted manually as recommended (Lauring et al., 2019). For the experiments in Supplementary Figures 1D–G (analysis of CP110 and TTBK2 presence on the MC), 3–4 fields of vision (200–400 cells) were analyzed per experimental run, *n* = 3. Statistical analyses by one-way ANOVA were performed using Graphpad Prism, *P* < 0.05 (^∗^), *P* < 0.01 (^∗∗^), *P* < 0.001 (^∗∗∗^), and *P* < 0.0001 (^****^). Results are presented as mean plus SEM. Primary antibodies used: Arl13b (Proteintech, Cat.no. 17711-1-AP), γ-tubulin (Merck, T6557), CP110 (Proteintech, 12780-1-AP), and TTBK2 (Merck, Cat.no. HPA018113).

### Dual Luciferase (TopFLASH) Assay, Transfection of HEK293

Transfection and dual luciferase assay of HEK293 WT and KO cells was carried out as previously described (Paclíková et al., 2017). In brief, in 0.1 μg of the pRLtkLuc plasmid and 0.1 μg of the Super8X TopFlash plasmid per well of 24 well plate were cotransfected, on the next day cells were treated by 90ng/ml WNT3a and signal was measured after 24 h treatment.

### CRISPR/Cas9 Generation of LRP5/6 Double Knock-Out and AXIN1/2 Double Knock-Out HEK293 Cells

Used guide RNAs were following: LRP5 gRNA gagcgggccgacaagactag, LRP6 gRNA ttgccttagatccttcaagt, AXIN1 gRNA cgaacttctgaggctccacg, and AXIN2 gRNA tccttattgggcgatcaaga. gRNAs were cloned into pSpCas9 (BB)-2A-GFP (PX458) (Addgene plasmid, 41815) or pU6-(BbsI)_CBh-Cas9-T2A-mCherry (Addgene plasmid, 64324) plasmids. Following transfection by Lipofectamine 2000 (Thermo Fisher Scientific) the transfected cells were FACS sorted [FACSAria Fusion (BD Biosciences)] and clonally expanded. Genotyping of LRP5 KO and AXIN2 KO mutants was done following genomic DNA isolation (DirectPCR Lysis Reagent; 301-C, Viagen Biotech) by PCR using DreamTaq DNA Polymerase (Thermo Fisher Scientific). Used primers: LRP5 forward: gttcggtctgacgcagtaca, LRP5 reversed: aggatggcctcaatgactgt, AXIN2 forward: cagtgccaggggaagaag, and AXIN2 reversed: gtcttggtggcaggcttc. PCR products were cut by BfaI (R0568S, NEB) in case of LRP5 KO and Hpy188III (R0622S, NEB) for AXIN2 KO screening, respectively. Successful disruption of individual ORFs was confirmed by sequencing, Supplementary Figures 2A,D, 3A–E.

## Results

### Treatment by Recombinant WNT3a Induces WNT/β-Catenin Pathway Activation but Not Ciliogenesis

**FIGURE 1:**
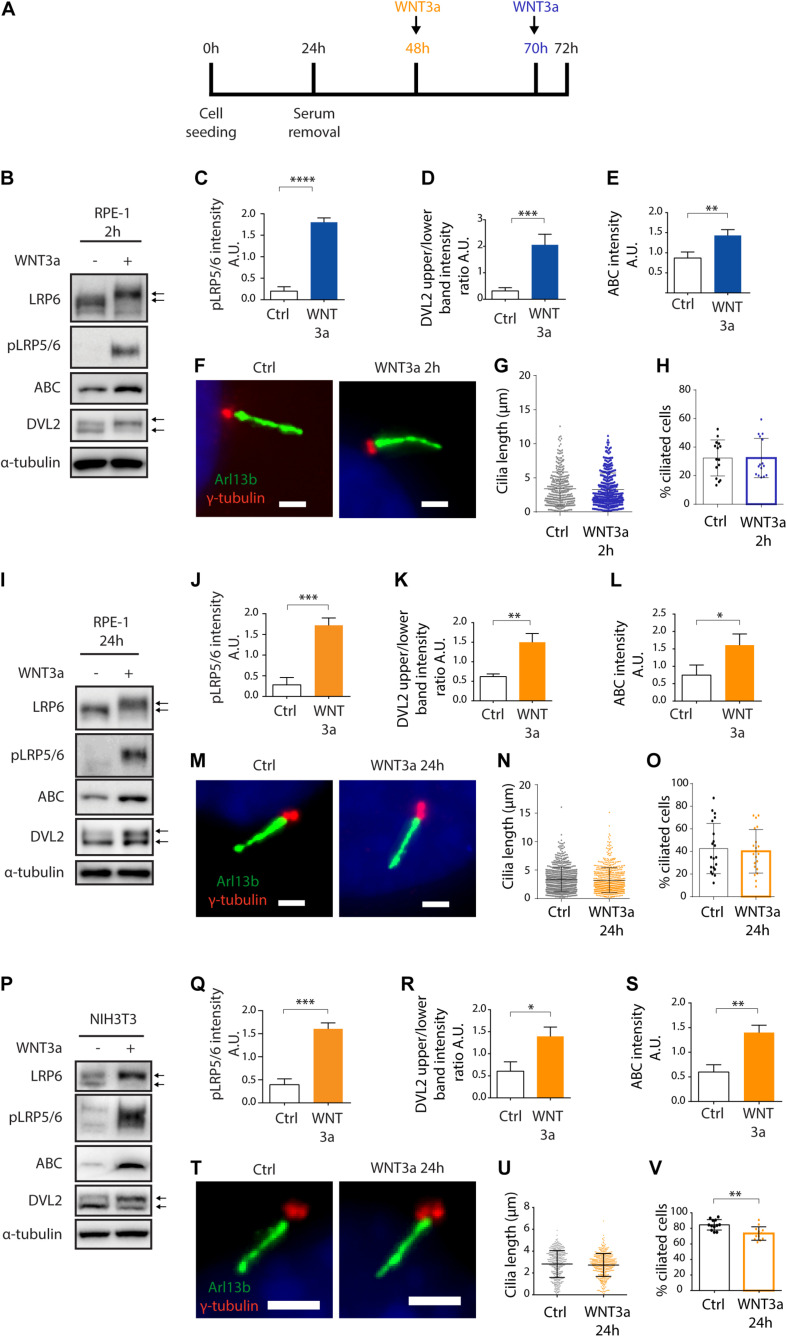
WNT3a does not promote ciliogenesis or cilia length. **(A)** Experimental scheme of WNT3a treatment experiment. Cells were seeded and grown for 24 h, then starved for additional 48 h. A 2 h treatment (RPE-1) by WNT3a is indicated in blue, 24 h treatment is indicated in orange (RPE-1 and NIH3T3). **(B)** Western blot analysis of 2 h WNT3a treatment of RPE-1. The treatment leads to LRP6 shift and increased LRP5/6 phosphorylation, DVL2 phosphorylation and upshift, and accumulation of ABC. The quantitation of pLRP5/6 intensity is shown in **(C)** *n* = 3, DVL2 band intensities (upper to lower band intensity ratio, the bands are indicated by arrows) is shown in **(D)** *n* = 3, the quantification of relative ABC levels is presented in **(E)** *n* = 3. **(F)** Representative images of RPE-1 cells treated by WNT3a or vehicle (control) for 2 h and stained for Arl13b (green) and γ-tubulin (red). Scale bar = 2 μm. DAPI (blue) was used to counter stain nuclei. The corresponding quantification of the cilia length **(G)** and the percentage of cells with Arl13+ cilium **(H)**. Each dot indicates either length of a single primary cilium **(G)** or percentage of ciliated cells in a single image **(H)**. **(I)** Western blot analysis of 24 h WNT3a treatment of RPE-1. The treatment leads to LRP6 shift, increased LRP5/6 phosphorylation, DVL2 phosphorylation and upshift, and accumulation of ABC. The quantification of pLRP5/6 intensity is shown in **(J)** *n* = 3, DVL2 bands (indicated by arrows) intensity ratio is shown in **(K)** *n* = 3, quantification of relative ABC levels is presented in **(L)** *n* = 3. **(M)** Representative images of RPE-1 cells treated by WNT3a or vehicle (control) for 24 h and stained for Arl13b (green) and γ-tubulin (red). Scale bar = 2 μm. DAPI (blue) was used to counter stain nuclei. The corresponding quantification of the cilia length and the percentage of cells with Arl13+ cilium is shown in **(N,O)**, respectively. Each dot indicates either length of a single primary cilium **(N)** or percentage of ciliated cells in a single image (O) *n* = 4. **(P)** Western blot analysis of NIH3T3 cells treated by WNT3a for 24 h shows LRP6 shift and LRP5/6 phosphorylation, DVL2 phosphorylation and upshift, and accumulation of ABC. The quantification of pLRP5/6 intensity is shown in **(Q)** *n* = 3, DVL2 band intensities (upper to lower band intensity ratio, the bands are indicated by arrows) is shown in **(R)** *n* = 3, quantification of relative ABC intensity **(S)** *n* = 3. **(T)** Representative images of NIH3T3 cells treated by WNT3a for 24 h, stained for Arl13b (green), and γ-tubulin (red). Scale bar = 2 μm. DAPI (blue) was used to counter stain nuclei. The corresponding quantification of the cilia length **(U)** and the percentage of cells with Arl13+ cilium **(V).** Each dot indicates either length of a single primary cilium **(U)** or percentage of ciliated cells in one image frame **(V)** *n* = 3.

First, we tested if primary ciliogenesis can be modulated by activation of WNT/β-catenin pathway in RPE-1 by recombinant WNT3a. Experiment outline is schematized (Figure 1A). We initially treated the cells for 2 h. While we observed the expected accumulation of active β-catenin (ABC), phosphorylation and shift of LRP5/6 coreceptors (LRP6, pLRP5/6, S1490/S1493), and phosphorylation and upshift of DVL2 (Figures 1B–E and Supplementary Figure 1A), WNT3a did not alter the length or number of Arl13b positive cilia (Figures 1F–H). Next, we examined effects of prolonged treatment of RPE-1 cells by WNT3a. Importantly, we were able to detect that WNT/β-catenin pathway is still active after 24 h, as visible from the mobility shift of LRP6 (Figure 1I and Supplementary Figure 1B) or the elevated levels of ABC, pLRP5/6 or DVL2 phosphorylation (Figures 1I–L), but the treatment did not show any notable effects on cilia length or numbers (Figures 1M–O). In agreement with these data, WNT3a treatment failed to alter either TTBK2 recruitment to MC (Supplementary Figures 1D,E) or MC-specific loss of CP110 (Supplementary Figures 1F,G). To corroborate these findings, we also tested the influence of WNT3a in NIH3T3 cell line. Similarly, to RPE-1, WNT3a treatment for 24 h was able to activate the WNT/β-catenin pathway in NIH3T3 cells (Figures 1P–S and Supplementary Figure 1C), but the length of cilia was not affected (Figures 1T,U). Intriguingly, we detected a decrease in the percentage of ciliated cells following the WNT3a treatment (Figure 1V).

### Inhibition of WNT Secretion Halts WNT Signaling but Not Ciliogenesis

**FIGURE 2:**
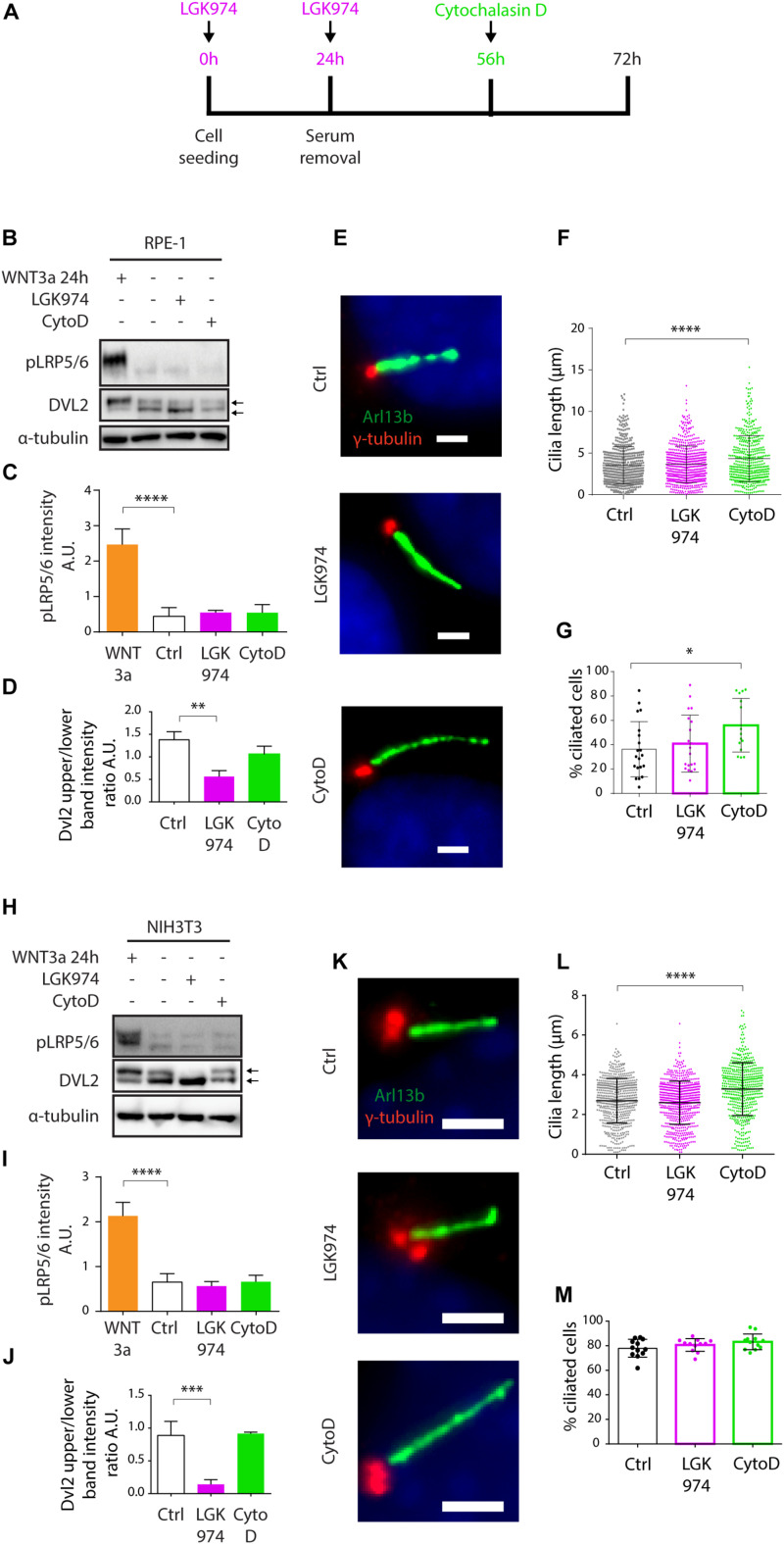
Inhibition of WNT secretion has no effect on ciliogenesis or cilia length. **(A)** Experimental scheme illustrating the time points of LGK974 (Purple) or CytoD (Green) treatments. **(B)** Western blot analysis of RPE-1 treated by LGK974 or CytoD. WNT3a was used as positive control to activate WNT/β-catenin pathway. pLRP5/6 intensity is quantified in **(C)** *n* = 3, DVL2 shift (upper to lower band intensity ratio) is quantified in **(D)** *n* = 3. **(E)** Representative images of RPE-1 cells following the indicated treatment, stained for Arl13b (green) and γ-tubulin (red). Scale bar = 2 μm. DAPI (blue) was used to counter stain nuclei. Quantification of the cilia length **(F)** and the percentage of cells with Arl13+ cilium **(G)**. Each dot indicates either length of a single primary cilium **(F)** or percentage of ciliated cells in one image frame **(G)** *n* ≥ 3. **(H)** Western blot analysis NIH3T3 treated by LGK974 or CytoD. pLRP5/6 intensity is quantified in **(I)** *n* = 3, **(J)** Quantification of DVL2 band intensities (upper to lower band intensity ratio) *n* = 3. **(K)** Representative images of NIH3T3 cells following treatment with LGK974 or CytoD, stained for Arl13b (green) and γ-tubulin (red). Scale bar = 2 μm. DAPI (blue) was used to counter stain nuclei. Quantification of the cilia length **(L)** and the percentage of cells with Arl13+ cilium **(M)**. Each dot indicates either length of a single primary cilium **(L)** or percentage of ciliated cells in one image frame **(M)** *n* = 3.

Having found WNT3a-activated WNT/β-catenin signaling is not sufficient to promote cilia formation, we tested a possibility that steady state WNT signaling is required for effective ciliogenesis. WNT/β-catenin pathway is intensively studied as a driver of oncogenic growth, thus there are currently available various small molecules that inhibit WNT ligand secretion. To this end, we used a Porcupine inhibitor LGK974 to block the secretion of endogenous WNT ligands and in turn block the steady state WNT signaling (Jiang et al., 2013). As a positive control in these experiments we used cytochalasin D (CytoD), an actin polymerization inhibitor known to facilitate ciliogenesis and promote cilia elongation (Kim et al., 2015). Experiment outline is schematized (Figure 2A). While we observed no visible change in pLRP5/6 levels following the LGK974 treatment (Figures 2B,C), perhaps because the basal levels of pLRP5/6 were at our detection limit, we detected downshift of DVL2 (Figures 2B,D) confirming the endogenous WNT signaling was successfully ablated. Importantly, however, the LGK974 treatment did not alter primary ciliogenesis, in contrast to CytoD that facilitated CP110 removal from MC (Supplementary Figures 1F,G), cilia elongation (Figures 2E,F), and formation (Figures 2E,G). In addition, we inhibited WNT signaling at the level of CK1-δ/ε using small molecule PF670462 (Badura et al., 2007; Janovska et al., 2018), and found no effect on ciliogenesis (Supplementary Figures 1H–J). Next, we applied the approach outlined in Figure 2A also to NIH3T3 cells, with very similar results - LGK974 caused no visible change in pLRP5/6 levels but inhibited WNT signaling on the level of DVL2 (Figures 2H–J), but LGK974 treatment failed to show any effect on cilia length, in contrast to CytoD treatment (Figures 2K,L). We noted the CytoD treatment in NIH3T3 did not increase the cilia numbers (Figure 2M), possibly due to high basal ciliation rate of NIH3T3 compared to RPE-1. In sum, these data imply that signaling mediated by endogenous WNT ligands is not required for primary ciliogenesis.

### Genetic Ablation of WNT/β-Catenin Pathway Does Not Alter Primary Ciliogenesis

To corroborate our findings, we established a panel of HEK293 cells devoid of critical components of WNT signaling pathways. To specifically block the course of WNT/β-catenin pathway we used LRP5/6 double knock out HEK293 cells, to block the course of any WNT signaling pathway we used DVL1/2/3 triple knock out HEK293 cells (Paclíková et al., 2017) and to overactivate WNT/β-catenin pathway we used AXIN1/2 double knock out HEK293 cells.

**FIGURE 3:**
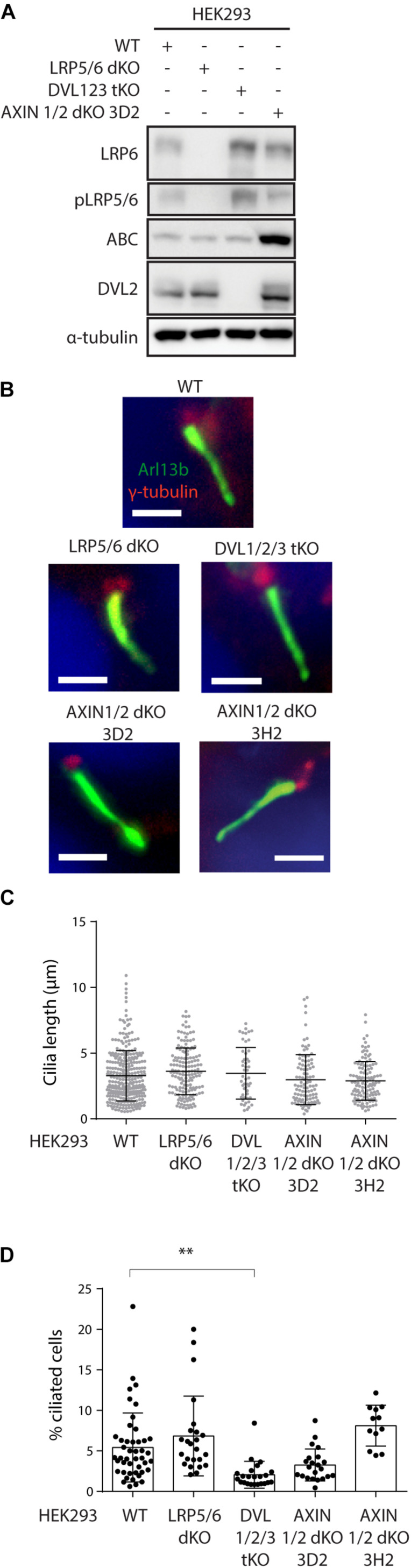
Ablation of WNT β-catenin pathway does not alter primary ciliogenesis. **(A)** Western blot analysis of individual HEK293 KO cell lines using the indicated antibodies. Note that LRP5/6 dKO cells lack LRP6 and phospho LRP5/6 (pSer1493/pSer1490), DVL1/2/3 tKO cell do not have detectable levels of DVL2. AXIN1/2 dKO cells shown elevated level of ABC. **(B–D)** HEK293 Cells were starved for 48 h, stained for Arl13b (green), γ-tubulin (red), and DAPI (blue), and analyzed by IF microscopy. Representative images are shown in **(B)**. Scale bar = 2 μm. Quantification of cilia length and percentage of ciliated cells is shown in **(C,D)**, respectively Each dot indicates either length of a single primary cilium **(C)** or percentage of ciliated cells in one image **(D)**. *n* = 4.

First, we have verified successful disruption of LRP5 gene by sequencing (Supplementary Figures 2A, 3A), and lack of LRP6 and pLRP5/6 signals in LRP5/6 null cells by western blot (Figure 3A and Supplementary Figure 2B). Furthermore, we confirmed these cells cannot activate WNT/β-catenin signaling (Supplementary Figure 2C). Similarly, we confirmed disruption of AXIN1 and AXIN2 genes in AXIN1/2 dKO by sequencing (Supplementary Figures 2D, 3B–E), and lack of AXIN1 by western blot (Supplementary Figure 2E). In addition, we observed that loss of AXIN1/2 function leads to excessive ABC accumulation (Figure 3A) and in turn to overactivation of WNT/β-catenin signaling in AXIN1/2dKO cells (Supplementary Figure 2F), as expected.

Having characterized our model system, we examined cilia formation in those cells. Consistently with previous work, HEK293 cells form cilia less frequently than RPE-1 or NIH3T3 cells (Lancaster et al., 2011; Bernatik et al., 2020). We were able to detect about 5% of cells with Arl13b+ primary cilium in WT HEK293. The percentage of ciliated cells, but not the cilia length, was reduced in DVL1/2/3 tKO cells (Figures 3B–D). This observation is in agreement with the role of DVL and WNT/PCP pathway in the regulation of basal body positioning and ciliogenesis (Park et al., 2008; Shnitsar et al., 2015; Sampilo et al., 2018). Systemic activation of WNT/β-catenin pathway by AXIN1/2 removal produced a somewhat mixed result. Using AXIN1/2 dKO clone 3D2 we initially observed a non-significant negative trend on the cilia formation. However, this was not confirmed using an independent clone 3H2 (Figure 3D). Importantly, the ablation of WNT/β-catenin pathway in LRP5/6 dKO cells had no effect on either the percentage of ciliated cells or cilia length (Figures 3B–D), in agreement with our earlier observations based on pharmacological inhibition of endogenous WNT signaling in RPE-1 or NIH3T3. In sum, from these data we conclude that WNT/β-catenin signaling is not required for effective ciliogenesis.

## Discussion

Regulation of ciliogenesis is a complex process involving multiple factors directly or indirectly influencing cilia initiation and elongation. The regulators of cilium formation encompass a wide range of molecules such as components of centrioles, regulators of vesicular trafficking, intraflagellar transport proteins, membrane proteins, and components of cytoskeleton (Seeley and Nachury, 2010; Ishikawa and Marshall, 2017; Wang and Dynlacht, 2018; Conkar and Firat-Karalar, 2020).

WNT3a is considered a prototypical “canonical” WNT ligand that activates WNT/β-catenin pathway (Willert et al., 2003). Moreover, WNT3a and hence the WNT/β-catenin pathway are well known for their mitogenic potential in many experimental systems (Niehrs and Acebron, 2012). In addition, WNT/β-catenin pathway has been shown to act mainly during G2/M phase of the cell cycle (Davidson et al., 2009), while primary cilia form during G0/G1 and during the G2/M they disassemble (Rieder et al., 1979; Ford et al., 2018). Furthermore, mitogenic signals typically promote cilium disassembly (Rieder et al., 1979; Tucker et al., 1979; Pugacheva et al., 2007). From this perspective, the recently reported positive role of WNT3a and WNT/β-catenin signaling on primary cilia formation (Kyun et al., 2020) is counterintuitive and puzzling.

Principally, there are several important methodological differences between our work and the previous results (Kyun et al., 2020) which may account for the different outcomes. (1) In our experiments we activated the WNT/β-catenin pathway by recombinant WNT3a, in contrast to WNT3a conditioned medium often used in the previous study (Kyun et al., 2020). Thus, some of the reported effects of WNT3a conditioned medium may be a result of secondary effects. (2) We applied up to 24 h stimulation by WNT3a to activate or 72 h LGK974 to block the pathway, respectively. We cannot formally exclude that the longer WNT3a treatments used by Kyun et al., could account for the observed differences. However, we argue this seems unlikely, given that full activation of the WNT/β-catenin pathway or cilium formation typically happens within several hours following the proper stimuli (Bryja et al., 2007; Naik and Piwnica-Worms, 2007; Pitaval et al., 2010; Lu et al., 2015; Wu et al., 2018; Pejskova et al., 2020). In fact, prolonged WNT/β-catenin pathway stimulation increases a chance for indirect secondary effects. Indeed, WNT signaling has been shown to regulate expression of a number of ligands from FGF (Kratochwil et al., 2002; Barrow et al., 2003; Shimokawa et al., 2003; Chamorro et al., 2005; Hendrix et al., 2006) or BMP (Baker et al., 1999; Kim et al., 2002; Shu et al., 2005) families that might in turn affect ciliogenesis (Neugebauer et al., 2009; Komatsu et al., 2011; Cibois et al., 2015; Bosakova et al., 2018). 3. Finally, we visualized cilia by staining for Arl13b, a small GTPase from Arf/Arl-family highly enriched in the ciliary membrane (Caspary et al., 2007; Cantagrel et al., 2008; Hori et al., 2008; Duldulao et al., 2009; Cevik et al., 2010; Li et al., 2010). In the report by Kyun et al., acetylated α-tubulin antibody staining was used to assess the cilia length, thickness, and numbers. From this perspective, it is plausible some of the reported changes in cilia length or thickness in fact reflect changes in the acetylation of ciliary tubulin rather than changes in cilium size. That being said, there is an evidence that individual cilia differ significantly in the levels of tubulin post-translation modifications and the levels of tubulin modifications may dramatically change in response to the appropriate stimuli (Piperno et al., 1987; Berbari et al., 2013; He et al., 2018).

Our data show that while WNT3a consistently activates the WNT/β-catenin pathway, it has no or minor negative effects on ciliogenesis. Elevated β-catenin levels following APC ablation have been related to reduced ciliogenesis and cell cycle defects in the developing cortex in mice (Nakagawa et al., 2017). Indeed, we detected modest decrease in the percentage of ciliated NIH3T3 cells following WNT3a induced β-catenin accumulation. We speculate we did not observe comparable negative effect on cilia following the WNT/β-catenin pathway activation after AXIN1/2 loss due to abnormal cell cycle regulation in HEK293, which hampers detection of relatively subtle deviations in their cell cycle progression (Löber et al., 2002; Stepanenko and Dmitrenko, 2015). These data are in contrast to Kyun et al., where accumulation of β-catenin by WNT3a conditioned medium treatment or by expression of S45A non-degradable oncogenic mutant variant of β-catenin (Liu et al., 2002) facilitates ciliogenesis.

In sum, we found no evidence that endogenous WNT/β-catenin signaling, while ablated either pharmacologically in RPE-1 or NIH3T3 by LGK974, or genetically by removal of LRP5/6 in HEK293, is required for primary cilia to form. Our findings presented in this article challenge some of the published evidence and argue against positive role of WNT3a or WNT/β-catenin pathway in ciliogenesis or cilia length regulation.

## Data Availability Statement

The original contributions presented in the study are included in the article/Supplementary Material, further inquiries can be directed to the corresponding author.

## Author Contributions

OB designed and performed the experiments, and wrote and edited the manuscript. PP and AK performed selection and verification of CRISPR edited HEK293 cell lines. VB edited the manuscript. LC designed the experiments, and wrote and edited the manuscript. All authors contributed to the article and approved the submitted version.

## Conflict of Interest

The authors declare that the research was conducted in the absence of any commercial or financial relationships that could be construed as a potential conflict of interest.
