# Supplementary material for: Primary Cilia Formation Does Not Rely on WNT/β-Catenin Signaling
Source: Front Cell Dev Biol. 2021 Feb 26;9:623753. doi: 10.3389/fcell.2021.623753 (PMC7952446; doi:10.3389/fcell.2021.623753)
Supplement: Supplementary file 1 [file Data_Sheet_1.PDF]

Supplementary Figure 1

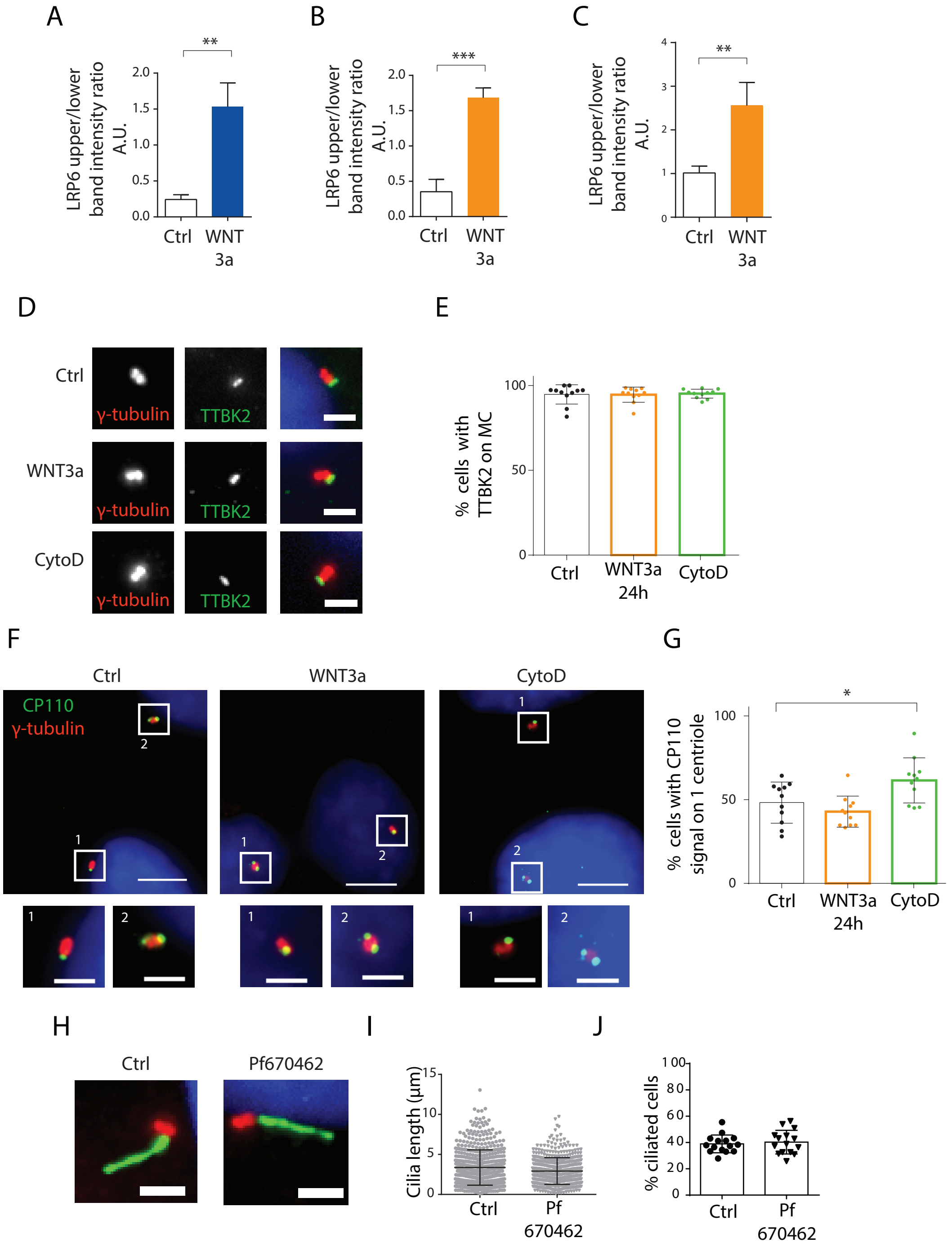

Supplementary figure 1: WNT3a does not affect cilia initiation in RPE-1

LRP6 upper to lower band intensity ratio as quantified for RPE1 treated by WNT3a for 2h (**A**), 24h (**B**) or NIH3T3 treated for 24h (**C**), n=3. RPE-1 cells were treated by either WNT3a or CytoD (see Fig.1A for details) and stained by the indicated antibodies. **(D)** Representative images of IF staining for TTBK2 (green) and γ-tubulin (red), DAPI (Blue) was used to counter stain nuclei, Scale bar 2μm. The quantification of a percentage of cells with TTBK2 signal at MC (one dot represents single image) is shown in **(E)** n=3. **(F)** Representative images of staining for centriole distal end protein CP110 (green) and γ-tubulin (red). DAPI (Blue) was used to counter stain nuclei. White rectangles indicate centrosomes with CP110 signal on either one centriole (1) of both centrioles (2), the insets are enlarged below. The effects of WNT3a or CytoD are quantified in **(G)** each dot represents a percentage of cells with CP110 signal present only on one of the two centrioles, n=3. Scale bar 5μm. Representative images of RPE-1 cells treated by Pf670462 or vehicle (control) for 24h and stained for Arl13b (green) and γ-tubulin (red) (**H**). Scale bar 2μm. DAPI (blue) was used to counter stain nuclei. The corresponding quantification of the cilia length (**I**) and the percentage of cells with Arl13+ cilium (**J**). Each dot indicates either length of a single primary cilium (I) or percentage of ciliated cells in a single image (J).

Supplementary Figure 2

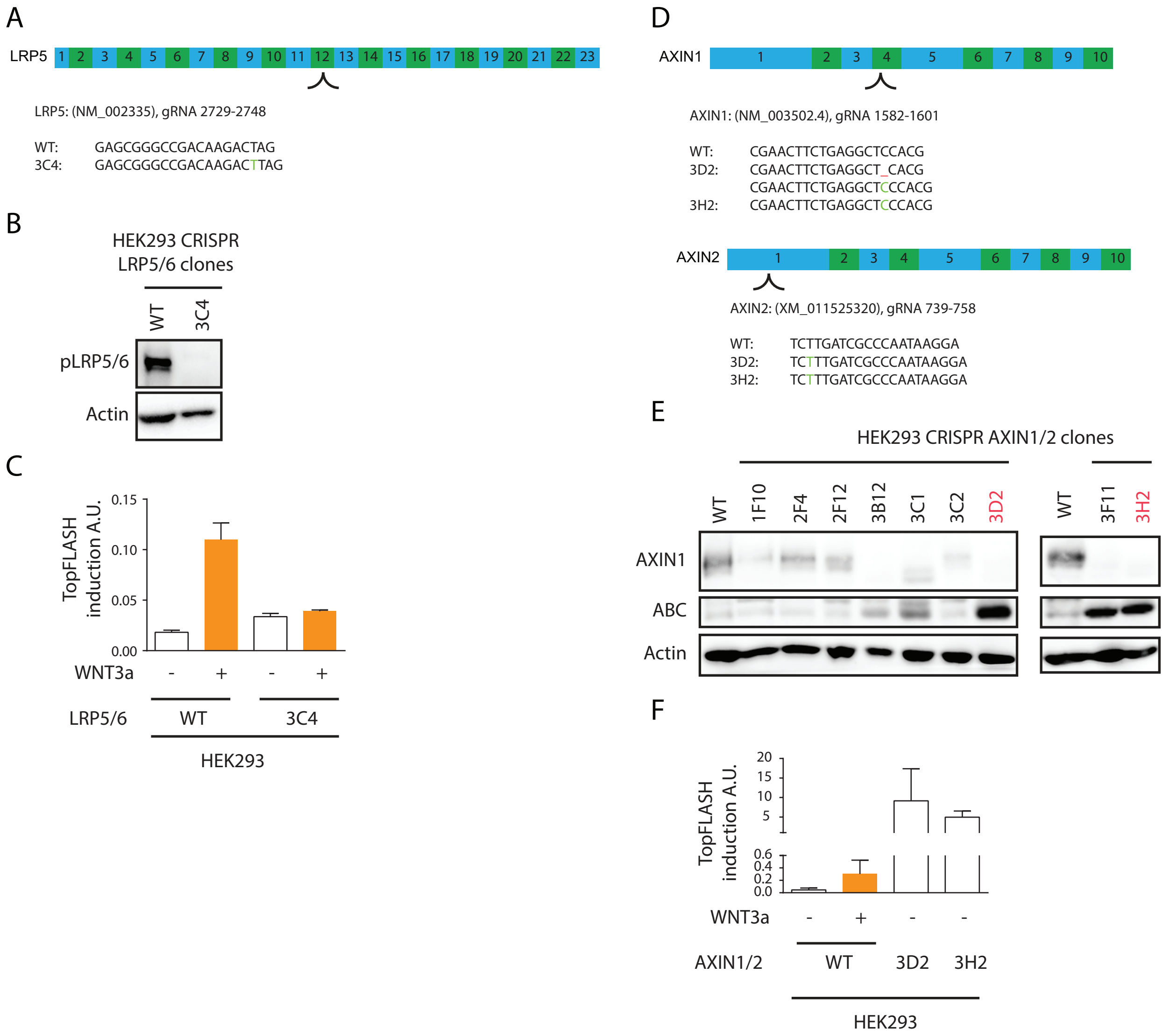

Supplementary figure 2: Verification of LRP5/6 and AXIN1/2 knock-out cells
(A) Scheme of LRP5 editing by CRISPR-Cas9. Individual exons are coded in blue and green. The used gRNA targets exon 12. Transcript variants unique identifier and exact position of gRNA are specified below. Cas9-edited sequence of LRP5 is indicated, the inserted "T" is highlighted in green. **(B)** Western blot analysis demonstrating the lack of phospho LRP5/6 signal in HEK293 clone 3C4 (LRP5/6 DKO). **(C)** Luciferase reporter assay showing that HEK293 clone 3C4 cannot activate WNT/β-catenin pathway. HEK293 WT and LRP5/6 dKO clone 3C4 were treated by WNT3a for 24h and activity of WNT/β-catenin pathway was measured by TopFLASH dual luciferase assay, n=3. **(D)** Schematic view of AXIN1 and AXIN2 editing by CRISPR-Cas9. Individual exons are coded in blue and green. Arrow indicates targeted exons for the used gRNAs, transcript variants unique identifiers and exact position of gRNAs are indicated below. Cas9-edited sequence of AXIN1 and AXIN2, detected indels are visualized in green and red, respectively. **(E)** WB detection of AXIN1 expression in the analyzed clones. Clones 3D2 and 3H2 which lacked AXIN1 expression and showed the highest levels of active β-catenin (ABC) were selected for further use. **(F)** Luciferase reporter assay showing that HEK293 clones 3D2 and 3H2 have overactive WNT/β-catenin pathway. Activity of the WNT/β-catenin pathway was measured by TopFLASH dual luciferase assay. 24h WNT3a treatment of HEK293 WT was used as positive control. Note that the reporter activity in untreated AXIN1/2 dKO clones 3D2 and 3H2 is notably elevated even if compared to WNT3a-treated WT cells.

Supplementary Figure 3

A

3C4 clone LRP5

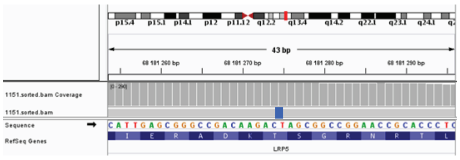

chr11:68 181 275, total count: 280, A : 0, C : 0, G : 0, T : 280 (100%, 140+, 140-), DEL: 0, INS: 269

B

3D2 clone AXIN1

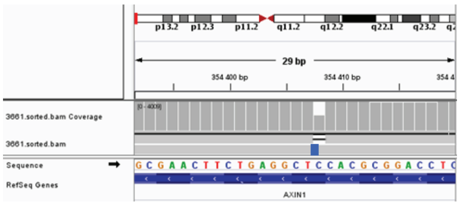

chr16:354 408, Total count: 2021, A : 1 (0%, 1+, 0-), C : 2017 (100%, 1012+, 1005-), G : 2 (0%, 1+, 1-), T : 1 (0%, 1+, 0-), DEL: 1936, INS: 1888

C

3D2 clone AXIN2

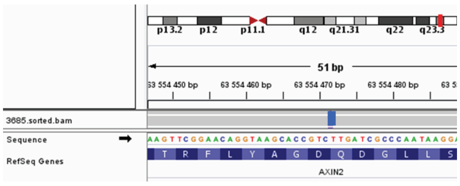

chr17:63 554 472, total count: 2267, A : 0. C : 1 (0%, 0+, 1-), G : 0, T : 2266 (100%, 925+, 1341-), DEL: 10, INS: 2032

D

3H2 clone AXIN1

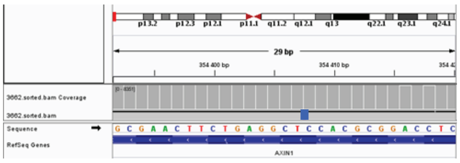

chr16:354 408, total count: 8038, A : 0, C : 8038 (100%, 3901+, 4137-), G : 0, T : 0, DEL: 13, INS: 7688

E

3H2 clone AXIN2

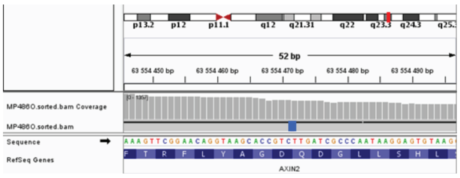

chr17:63 554 472, total count: 940, A : 0,C : 0, G : 0, T : 940 (100%, 387+, 553-), DEL: 0, INS: 917

Supplementary figure 3: Sequencing of HEK293 CRISPR KO clones 3C4, 3H2 and 3D2
Sequencing of clone 3C4 for LRP5 (A) and clones 3D2 for AXIN1 (B) and AXIN2 (C), clone 3H2 for AXIN1 (D) and AXIN2 (E). Exports from Integrative Genomics Viewer (igv.org), red marker on the chromosome map shows position of the enlarged area, exact position is defined by nucleotide numbers below. The blue mark above the reference sequence indicates the change detected in the read compared to the reference sequence. The description includes nucleotide number, total number of reads, type of nucleotide detected, + and - defines read strand. DEL indicates deletion, INS indicates insertion. Red color highlights resulting mutation and the type.
